# Approaches to the development of new screening tools that assess distress in Indigenous peoples: A systematic mixed studies review

**DOI:** 10.1371/journal.pone.0291141

**Published:** 2023-09-08

**Authors:** Kathryn Meldrum, Ellaina Andersson, Valda Wallace, Torres Webb, Rachel Quigley, Edward Strivens, Sarah Russell

**Affiliations:** 1 College of Medicine and Dentistry, James Cook University, Cairns, Queensland, Australia; 2 Monash Children’s Hospital, Clayton, Victoria, Australia; 3 Queensland Health, Cairns and Hinterland Hospital and Health Service, Cairns, Queensland, Australia; Walden University, UNITED STATES

## Abstract

This mixed studies review assessed the extent of the literature related to approaches used to develop new tools that screen for distress in Indigenous adults globally. It answered the research question: What qualitative and quantitative approaches are used to develop new screening tools that assess distress in Indigenous peoples globally? CINAHL, Embase, Emcare, Medline, PsychInfo and Scopus databases were systematically searched to identify relevant articles published between January 2000 and February 2023. Articles describing the development of a new screening tool for Indigenous peoples, globally, published in English since 2000 and constituted a full publication of primary research, met the inclusion criteria. Studies underwent quality appraisal using the Mixed-Methods Appraisal Tool. A sequential exploratory design guided data analysis. Synthesis occurred using a two-phase sequential method. Nineteen articles constituted the data set. Articles described the use of qualitative, quantitative, or mixed methods in approximately equal numbers. Overall, qualitative methods were used in early stages of tool development, with mixed and quantitative methods used to pilot and validate them. However, most studies did not follow the theoretical guidelines for tool development, and while validation studies took place in over half of the data set, none adequately assessed construct validity. Sixty percent of the articles were located using citation searches, which suggests database searches were ineffective. Valid tools that screen for distress in Indigenous populations support equitable access to health care. This review found that most screening tools were developed in Australia. However, additional evidence of their validity is needed in addition to a valid diagnostic tool that supports the determination of criterion validity. These needs present important future research opportunities.

## Introduction

Indigenous peoples globally “retain social, cultural, economic, and political characteristics that are distinct from the dominant societies in which they live” [1, p. 1]. Their worldviews are interconnected and interrelated with those of their community and the environment. They also have a holistic view of their wellbeing [2] which encapsulates mental, physical, psychosocial and spiritual aspects [3–6]. Indigenous people use different terms to describe their wellbeing [6]. For example, American Indians use the term wellness [5], Australian Aboriginal and Torres Strait Islander peoples, social and emotional wellbeing [4], and Canadian First Nations peoples use different terms depending on their cultural affiliation [6]. Despite the different terminology, all share the holistic characteristics of wellbeing that are indivisible from each other.

### Screening for distress in Indigenous peoples

Historical and continuing impact of colonialism negatively impact the wellbeing and health outcomes of Indigenous peoples. Notwithstanding these issues, Indigenous peoples are often positioned as in deficit with respect to their health and their concomitant outcomes [7]. One example of continued impact of colonialism is the use of screening tools designed to assess distress in Indigenous peoples, specifically depression and anxiety, that have been developed using the dominant Western biomedical model [8–10]. Screening tools are used to identify signs of distress so that people can be referred for diagnosis and treatment [11]. A recent scoping review [12] found that the Patient Health Questionnaire– 9 (PHQ-9), Centre for Epidemiological Studies Depression Scale (CES-D) and Kessler Psychological Distress Scale (K10, K6, K5) were still the most used screening tools to assess distress in Indigenous peoples globally. While some cross-cultural adaptation had taken place, many of these tools were not validated with the populations that they were being used with. The outstanding recommendation from the scoping review was that more work needed to be done to support the needs of Indigenous peoples by investigating how new tools were developed and validated with and for them.

Indigenous peoples’ worldviews and holistic view of their wellbeing contrast with that of the dominant western biomedical model [3]. Many authors [4, 13–16] have identified that decolonising psychology needs to occur by “removing the impacts of historical domination on subordinated populations by powerful outsiders” [17, p. 259]. Decolonising psychology can be achieved by recognising different worldviews [18] and incorporating Indigenous peoples’ cultural perspectives and practices into service provision and research [4, 16]. As part of a commitment to decolonising psychology screening tools that embody Indigenous peoples’ holistic conceptualisations of wellbeing need to be developed [19–21] to ensure that appropriate treatment can be accessed.

### Background

As described in the protocol associated with this systematic review [22], the context of this work is centred on the development of a new tool to support screening for distress in Torres Strait Islanders living in the Northern Peninsula Area (NPA) of Australia and Torres Strait Islands (Zenadth Kes) using the decolonised conceptualisation health and wellbeing, social and emotional wellbeing [4, 13, 23]. The need for this work emanated from a dementia prevalence study conducted in the NPA and Torres Strait [24–26] that used mainstream screening tools for depression and anxiety. Both screening tools were found to be inappropriate for use with this population [26]. Therefore, and in response to community and health practitioner feedback, a project to develop an appropriate screening tool was instigated.

### Rationale

#### Objective and research question

The objective of this systematic mixed studies review (SMSR) was to assess the extent of the literature related to approaches used to develop new tools to screen for distress in Indigenous adults. The overarching research question guiding the review was: What qualitative and quantitative approaches are used to develop new tools to screen for distress in Indigenous adults globally? Sub-questions included: 1) What are the different approaches for developing new tools?; 2) How do qualitative, quantitative, and mixed methods interact in the development approach?; 3) Do subsequent tools demonstrate validity, reliability, and acceptability for the target population?; and 4) Is there an overarching development approach? For this SMSR, new tools were any screening tool that has been adapted in any way (language translation or cross-cultural) from a standard (Western) (hereafter referred to as standard) tool, as well as those developed with Indigenous peoples’ involvement in their conceptualisation and design. The focus of this review was specifically on tools that screen for low mood and/or anxiety as well as those that screen for Indigenous conceptualisations of distress.

## Materials and methods

This SMSR was conducted as described in the protocol [22] and guided by the eight-stage method proposed by Pluye et al., [27]. The protocol was not registered. The eight-stage method [27] included: 1) Determine review question; 2) Determine eligibility criteria; 3). Establish information sources; 4) Identify potentially relevant studies; 5) Select relevant studies; 6) Appraise study quality; 7) extract data; and 8) synthesis included studies.

### Changes made after the protocol was published

After the publication of the protocol, three changes were made to support analysis and synthesis of the data. First, in sub-research question 3 the term “clinical utility”. was replaced with “the evaluation of a tool as it is used in a clinical setting post-development” [28], as this was more suited to phase one analysis.

Second, the development of the conceptual model and refinement of the method for analysis and synthesis of the data, including the adoption of definitions for methods used for validation of tools, were made after protocol publication. Finally, a quality criteria framework developed by Terwee and colleagues [29] and adapted by Schellingerhout and colleagues [30] was used to evaluate the findings from phase two, quantitative analysis. Consequently, additional data analysis and synthesis details not provided in the protocol for this review are detailed the sections headed Stage 7 and 8 below. This SMSR is reported according to the Preferred Reporting Items for Systematic Review and Meta-analysis (PRISMA) 2020 statement and checklist [31].

### Stage 7 –Extract data

A sequential exploratory design was used to extract data from the studies [32, 33]. Phase one was focussed on extracting qualitative data from all studies. In addition to identifying the approach to developing new tools, each tool was categorised into one of four groups. Tool type categories included: 1) language translation of a standard; 2) cross-cultural adaption of a standard tool; 3) tools with both standard and Indigenous designed scales; and 4) Indigenous designed only. During this data extraction phase, studies that also used quantitative methods were identified and subsequently analysed in phase two.

Phase two focussed on extracting quantitative data by counting approaches to establishing the reliability and validity of new tools as well as extracting quantitative data from studies that contained it. In addition to citation headings, the Excel spreadsheet included methods used to validate and/or establish the reliability of the new tool. Validity methods included in the data extraction spreadsheet were: 1) Content; 2) face (acceptability); 3) construct; 4) convergent; 5) divergent; 6) known group; 7) concurrent; 8) predictive; 9) sensitivity (%); 10) specificity (%); 11) positive predictive values (PPV) (%); and 12) negative predictive values (NPV) (%). Methods for establishing tool reliability listed in the spreadsheet included: 17) Internal consistency; 18) Test re-test; 19) Inter-rater reliability. When any of the methods were identified in studies, they were recorded in the spreadsheet with a 1 in the associated column. The associated numerical values from statistical analysis related to validity and/or reliability from each of the studies were recorded in a separate table. Data from each study was systematically and independently extracted by two reviewers (KM and SR).

To support phase two data extraction and subsequent analysis, definitions of the measurement properties their domains, aspects and sub-aspect(s) of their properties (where relevant) were agreed upon. Measurement properties for this systematic review were divided into two domains: reliability and validity. For the purposes of this SMSR reliability was defined as a tool that consistently produces the same result across time (test re-test), assessors (inter-rater) or across questions (internal consistency) [34]. Validity was defined as a tool that captures the attribute being measured [35]. Additionally, a reference or gold standard is a test/instrument that determines whether a person has the target condition [36]. For screening tools, a reference standard is a diagnostic interview [37].

Any type of psychometric tool needs to satisfy basic properties to be used [35]. Reliability and validity are basic properties. Tools need to be reliable and valid otherwise there is a risk that incorrect or biased results will lead to a wrong conclusion [38]. Consequently, tools need to be developed and validated cautiously and robustly to avoid bias.

### Conceptual framework

The development of new tools and adaption of previously developed ones are critical to supporting the health and wellbeing of people across the world [39]. Tool development is not an easy task [40] and there is significant variation in approaches to it [39] making it time-consuming and resource intensive. To enable a comparison between the theoretical literature and published studies describing tool development, a conceptual framework was developed.

#### Development of conceptual framework

**Fig 1:**
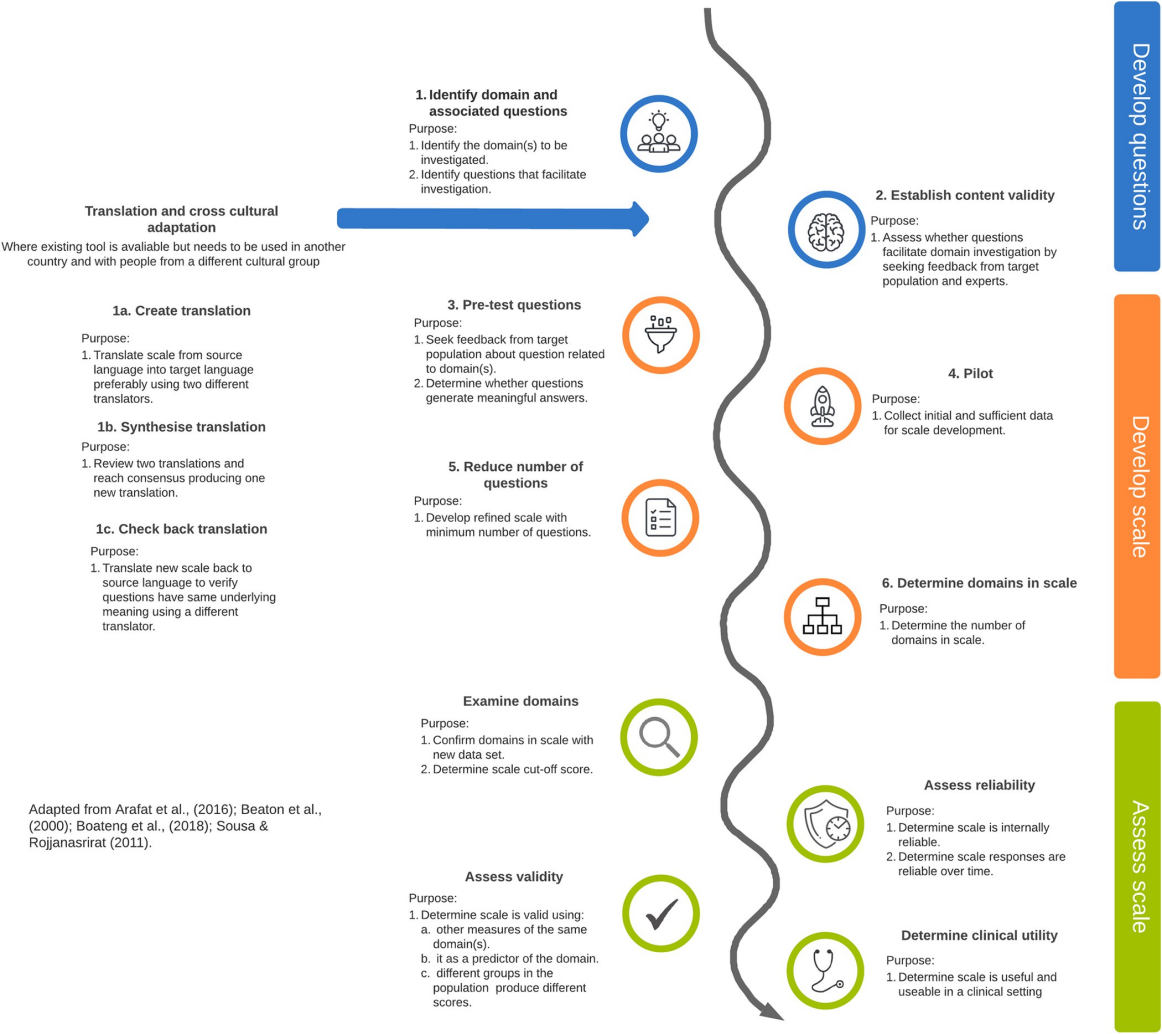
Questionnaire development conceptual framework (adapted from [39–42]).

The conceptual framework presented in Fig 1 was adapted from a range of key resources [39–42]. Ideally the primer written by Boateng and his colleagues [40] would have met the needs of this review but did not include the steps for cross-cultural adaptation, important to this review. The work of Beaton and her colleagues [42] on the cross-cultural adaptation of self-report measures is well cited and supported by more recent work [39]. This literature met the needs of the review but did not incorporate steps needed to validate new tools. Consequently, baseline development and cross-cultural adaptation processes were incorporated into the conceptual framework for this review. To make it easier to use the conceptual framework for analysis and synthesis of the data in the review, only the purpose of each of the steps were outlined.

### Determining quality of measurement properties

In addition to measuring the overall quality of the papers using the Mixed Methods Appraisal Tool (MMAT), findings of the studies included in phase two were assessed according to the criteria proposed by Terwee et al., [29] and adapted by Schellingerhout et al., [30] (S1 Table). This process was completed because simply identifying what approaches were used and their associated numerical values did not provide any indication of the quality of reliability and validity findings.

The quality of the predictive capacity of new tools is absent from the quality criteria (S1 Table). Consequently, we calculated a weighted diagnostic odds ratio (DOR) according to the method described by Glas et al., [43] for each paper that reported the outcomes for predictive validity. The quality criteria applied by Ali et al., [37] of DOR > 50 = very strong, between 50–20 = strong, between 20–10 = fair and <10 weak was utilised to classify each tool’s predictive capability.

### Stage 8 –Synthesise included studies

The findings from qualitative phase one analysis were mapped on to the questionnaire development conceptual framework adapted for this study (Fig 1). In addition, to determine whether there was a correlation between the quality of the studies determined by the MMAT and number of steps authors took to develop their tool, Spearman’s rho was calculated using the Statistical Package for the Social Sciences (SPSS) (version 28.0.1). Where tool development was reported over several publications, for example the adaptation of the PHQ-9 [44–47], a mean MMAT score was calculated for all related publications. If the mean MMAT was a fraction it was rounded up or down to the nearest whole number. As MMAT scoring was ordinal it was converted to a dichotomous score. Consequently, MMAT scores between 0–2 were coded 1 (low) and scores 3–5 were coded 2 (high). The findings from this phase answered sub-research questions one and two.

The quantitative phase two analysis identified the number of studies using a range of methods available for determining validity and/or reliability of the new tool with the target population. Quantitative data about the reliability and validity of the tools was also extracted and evaluated using a quality framework [29, 30]. Meta-analysis of this data was not conducted due to the heterogeneity of the study designs. Findings of this phase were used to answer sub-research question three. Finally, findings from phases one and two were synthesised to answer sub-research question four.

## Results

**Fig 2:**
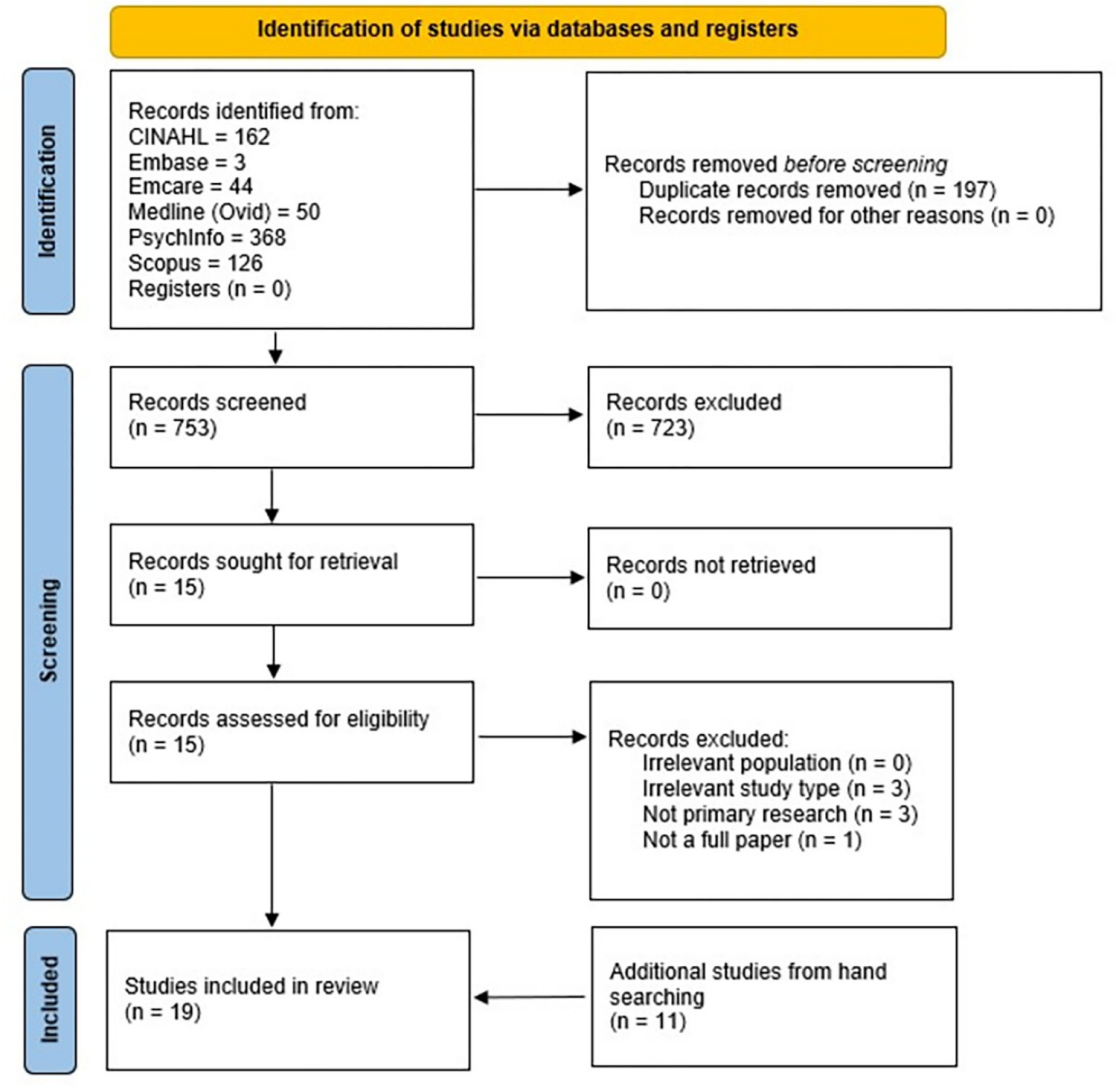
PRISMA flow diagram for this systematic mixed studies review [48].

Seven hundred and fifty-three (753) records were retrieved from database searches and imported into Endnote. After titles and abstracts of potential records were independently reviewed by two authors (KM and EA), 723 records were excluded. Interrater reliability was 0.67 (p < 0.011), (Kappa Measure of Agreement) which was at the higher end of moderate agreement (e.g. 0.5–0.7) [45]. Disagreements between reviewers were discussed until consensus was reached. Subsequently, 15 full text records were independently assessed for eligibility against the inclusion criteria by two reviewers (KM and SR). After excluding seven records, eight remained. A further 11 records were obtained from hand searches (Fig 2). Consequently, 19 studies were included in this SMSR. Fig 2 illustrates the PRISMA [48] flow diagram for this SMSR. Full search strategies for each database are detailed in supporting information 1 (S1 File).

### Demographics of the data set

All 19 studies were published between 2007 and 2021, with 14 since 2010. The most publications in any one year was three (2017; 2019). Most studies were undertaken in Australia (n = 10) with single studies in Canada and India. These numbers do not add up to19 because related studies published by the same authors, for example Brown et al., [44] Brown et al., [45] Getting it Right Collaborative [46] and Farnbach et al., [47] were grouped together.

Eight studies [44–47, 49–52] had at least one author who identified as a First Nations person of the country where the study was conducted. This information was either provided in the article or the authors are known to identify as a First Nations person.

The overarching design adopted for each of the studies was almost evenly spread with seven studies adopting qualitative and quantitative methods, respectively and five studies adopted mixed methods. Nine studies were undertaken with participants recruited from a primary health care setting and seven studies from a community setting only. Three studies took place with participants recruited from both primary care and community settings and one in both acute and primary care settings. Table 1 below identifies which papers were classified into each category and the tools used in their development.

**Table 1:** The classification of each paper (n = 19) and screening tools used in their development.

Tool type			
Category 1. Language translation Tool (n = 1) [citation]	Category 2. Cross-cultural adaptation Tool developed (n = 12) [citation]	Category 3. Standard and Indigenous Tool developed (n = 3) [citation]	Category 4. Indigenous Tool developed (n = 2) [citation]
EPDS [53] (dep & anx)	PHQ-9 [44, 46, 54–56] (dep)	GEM + K6 [51] (emp, dep. & anx.)	HANAA [20, 61] (SEWB)
	DASS-21 [57] (dep & anx)	Psychosocial Questionnaire + aPHQ-9 + K6 [45] (dep & anx.)	PANAS [62] (positive and negative affect)
	EPDS [52, 58–60] (dep & anx)	adapted GEM [49] (emp, dep. & anx.)	
	K5 [50] (dep. & anx.)		

Abbreviations used in Table: Edinburgh Postnatal Depression Scale (EPDS); Depression (dep.); Anxiety (anx.); Patient Health Questionnaire– 9 (PHQ-9); Depression Anxiety and Stress Scale (DASS-21); Kessler Psychological Distress Scale—6 (K6); Growth and Empowerment Measure (GEM); Empowerment (emp); Here and Now Aboriginal Assessment (HANAA); Social and Emotional Wellbeing (SEWB); Positive and Negative Affect Scale (PANAS).

### Quality appraisal

The MMAT [63] was used to appraise the quality of each of the studies in the data set. It has five elements for each category and foregrounds the need for a research question(s) to be included in empirical studies as they underpin the rationale for study design. Each category begins with a question that relates to the research question. In rating each paper, a point was allocated to each MMAT component identified in the study. Consequently, the highest quality papers scored 5 points and the lowest 0. Critical appraisal revealed that only one paper [59] of 19 articulated a research question and was the only one that scored 5/5. Seven papers contained three positive components, five two, three one, and three zero. Consequently, most of the papers of lower quality according to MMAT ratings. Table 2 summarises the main characteristics of the included studies and illustrates how each study was rated on the MMAT.

**Table 2:** Main characteristics of studies included in this SMSR.

First author [citation]	Publication year	Country	Study design	Setting (A–acute; C–community; PH–primary health)	Intended tool use	Tool type (1–4)^a^	Participants^b^	Mean age (range years)	MMAT quality score
Almeida [56]	2014	Australia	Quantitative descriptive	C	Clinical	2	250	60.9 ± 10.7 (46–89)	1
Brinckley [50]	2021	Australia	Mixed methods	C	Research	2	6988	(<16)	3
Brown [44]	2013	Australia	Qualitative	C	Clinical	2	Not applicable		3
Brown [45]	2016	Australia	Mixed methods	C	Clinical	3	186	38.9 ± 12.5 (16–72)	0
Campbell [53]	2008	Australia	Quantitative descriptive	PH	Clinical	1	210		2
Carlin [58]	2019	Australia	Qualitative	C	Clinical	2	15	(8–42)	3
Carlin [52]	2020	Australia	Qualitative	C	Clinical	2	18		3
Esler [54]	2007	Australia	Qualitative	PH	Clinical	2	33^c^		3
Esler [55]	2008	Australia	Quantitative descriptive	PH	Clinical	2	34	57.7	1
Farnbach [47]	2019	Australia	Mixed methods	PH		2	40		2
Getting it Right Collaborative [46]	2019	Australia	Quantitative descriptive	PH	Clinical	2	500	43 ± 15 (18–80)	2
Gomez Cardona [49]	2021	Canada	Qualitative	C	Clinical	3	12 Canadian First Nations and non-Indigenous		0
Haswell [51]	2010	Australia	Quantitative descriptive	C; PH	Research	3	184	39.9	2
**First author [citation]**		**Country**	**Study design**	**Setting**		**Tool type (1–4)** ^**a**^	**Participants** ^**b**^	**Mean age (range years)**	**MMAT quality score**
Janca [20]	2015	Australia	Mixed methods	A; PH	Clinical	4	30	37 (18–63)	3
Janca [61]	2017	Australia	Quantitative descriptive	PH		4	38		1
Kotz [59]	2016	Australia	Qualitative	PH; C	Clinical	2	172		5
Marley [60]	2017	Australia	Mixed methods	PH	Clinical	2	97		3
Schlesinger [57]	2008	Australia	Quantitative descriptive	PH; C	Clinical	2	175	35 ± 11.5	0
Snodgrass [62]	2017	India	Mixed methods	C	Research	4	219 Indigenous Indians		2

^a^ 1) Language translation 2) Cross-cultural adaptation 3) Standard and Indigenous 4) Indigenous.

^b^ Aboriginal and/or Torres Strait Islander participants unless otherwise identified.

^c^ 30 identified as being either of Aboriginal or Torres Strait Islander descent

### What are different approaches for developing new tools?

The results of phase one, qualitative analysis, were mapped against the conceptual model adapted for this SMSR. Table 3 illustrates the range of approaches taken by each study.

**Table 3:** Phase one qualitative analysis mapped against the conceptual model.

	Activity	Method	First author (citation)	
1. Identify domain and associated questions	Identify lexicon	Focus groups or interviews	Janca [20] Snodgrass [62]	Develop questions
	Develop conceptual model	Literature review + thematic analysis of previously conducted interviews	Brown [45]	
	Develop domains	From lexicon	Janca [20] Snodgrass [62]	
		Interviews + literature review + expert review + focus group	Brown [44]	
	Identify previously developed tool	Focus groups	Brown [44] Kotz [59] Gomez-Cardona [49]	
Translation and cross-cultural adaptation	1a) Create translation	Focus groups	Brown [44]	
	1b) Synthesise translation	Focus groups	Brown [44]	
	1c) Check back translation	Focus groups	Brown [44]	
2. Establish content validity		Focus groups	Esler [54] Schlesinger [57] Campbell [53] Brown [44] Almeida [56] Janca [20] Kotz [59] Carlin [58]	
3. Pre-test questions		Focus groups	Janca [20] Brown [44] Kotz [59] Snodgrass [62]	Develop scale
4. Pilot		Deploy questionnaire	Janca [20] Brown [45] Snodgrass [62]	
5. Reduce number of questions		Statistical analysis	Schlesinger [57] Haswell [51]	
6. Determine domains in scale		Statistical analysis	Schlesinger [57] Haswell [51] Brown [45] Snodgrass [62]	
Examine domains^a^	Determine tool cut-off score (sensitivity, specificity, PPV/NPV)	Receiver operating characteristics (ROC) curve analysis	Esler [55] Almeida [56] Marley [60] Getting it Right Collaborative [46] Brinckley [50]	Assess scale
Assess reliability		Deploy questionnaire	Schlesinger [57] Campbell [53] Esler [55] Haswell [51] Almeida [56] Janca [20] Brown [45] Marley [60] Snodgrass [62] Getting it Right Collaborative [46] Brinckley [50]	
Assess validity		Deploy questionnaire	Schlesinger [57] Campbell [53] Esler [55] Haswell [51] Almeida [56] Janca [20] Brown [45] Marley [60] Snodgrass [62] Getting it Right Collaborative [46] Brinckley [50]	
Evaluate clinical use		Interview/ questionnaire	Janca [61] Farnbach [47] Carlin [52]	

^a^These steps are not numbered because they may take place in any order and/or concurrently.

Establishing tool reliability and validity were completed an equal number of times (12). Overall, Brown and colleagues [44–47] completed the most steps (11) to adapt, develop the scale, examine the reliability, validate, and evaluate the clinical use of the PHQ-9 for Australian First Nations peoples. These steps also included the development of a category three tool which used a mix of Indigenous developed and standard tools (aPHQ-9 and K6) [45]. Kotz and colleagues [52, 58–60] Snodgrass and colleagues [62] and Janca and colleagues [20, 61] completed eight steps to develop the Kimberly Mums Mood Scale (KMMS), the PANAS and HANAA, respectively. The KMMS developers did not report the translation and cross-cultural adaptation steps advocated by Beaton et al., [42]. Consequently, only three steps were recorded in the first phase of the conceptual model. The PANAS and HANAA are category 4 (Indigenous tools) which did not need to progress through translation steps. The creators of the PANAS have reported reliability and validity, but not clinical use of the screener. Whereas the creators of the HANAA have reported reliability, validity, and its clinical use.

Other tool adaptations have completed comparatively fewer steps with Schlesinger et al. [57] reporting five and Almedia et al., [56], Esler et al., [54, 55] and Haswell et al., [51] four. Both Brinkley et al., [50] and Campbell et al., [53] only completed three steps. Campbell et al., [53] did not report having undertaken the translation and cross-cultural adaptation steps advocated by Beaton et al., [42]. However, it is noted that community consultation and participatory action research approaches were used during translation, so perhaps these steps were undertaken but not subsequently published. These authors only completed one-step in phase one and two in phase three. In contrast, Brinckley et al., [50] only intended to validate a previous adaption of the K5, so completed three steps in phase three. Gomez-Cordona et al., [49] only reported completing one step in phase one to adapt the GEM to the Canadian context.

The relationship between MMAT score and number of steps to develop the tool was investigated using Spearman’s rho (see S2 Table). There was a medium, [64] positive correlation between the two variables, (*r* = 0.35, n = 11, *p* = 0.29). The small sample size reflects the low *p* valve.

### How do qualitative, quantitative, and mixed methods interact in the development approach?

According to the conceptual model, phase one–develop questions uses qualitative methods. These methods include literature reviews, expert reviews, interviews, and focus groups. Phase two uses mixed methods such as focus groups as well as exploratory (EFA) and confirmatory (CFA) factor analysis. The final phase–assess scale, primarily uses quantitative methods. However, more recently authors [47, 52, 61] have started to evaluate the clinical use of newly developed tools. Mixed methods have been employed to conduct these evaluations.

**Fig 3:**
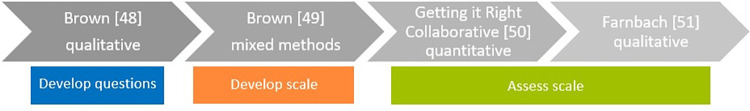
Alignment of the adaptation of the PHQ-9 with the conceptual model.

The author’s [44–47] approach to publishing the adaptation of the PHQ-9 aligns with the conceptual model. Fig 3 below illustrates this.

The adaption of the EPDS to the KMMS [52, 58–60] followed a similar pattern. However, the two later papers [52, 58] used qualitative methods such as focus groups and interviews. In contrast, Janca et al., [20] described six of the seven steps they used to develop the HANAA in one publication. Their later publication [61] outlined the clinical use of the HANNA, which was determined using an online survey. The analysis method was not reported, however, numerical scores were provided for some questions, so it is presumed that quantitative methods were employed. Themes were also reported. However, it is unclear whether these were derived from summing question responses or from thematic analysis of text-based open-ended responses. From a conceptual perspective, there is a methodological path to guide new tool design with qualitative approaches more suited to the first phase, mixed methods for the second and quantitative for the third. Nevertheless, authors decide on a publication plan which may differ from the theoretical approach.

### Do tools demonstrate validity, reliability, and acceptability for the target population?

The findings presented below are a result of phase two—quantitative analysis of data. Eleven papers [20, 45, 46, 50, 51, 53, 54, 56, 57, 60, 62] were included in this analysis.

Initially, Table 4 presents a quantitative sum of the number of studies investigating the reliability and validity of their screening tool. Subsequently, Table 5 presents the quantitative findings of reliability and validity of the newly developed tools. Finally, quality criteria [29, 30] were applied to the reliability and validity findings to determine whether they were robust. These findings are presented in Table 6.

**Table 4:** Quantitative sum of number of studies investigating reliability and validity of screening tools.

	Reliability			Validity						
	IC^a^	TR^b^	IR^c^	Content	Construct				Criterion	
					Structure	KG	Discrim.	Converg.	Concur.	Predict.
Number of studies	10	1	1	9	5	1	1	5	7	6

^a^Internal consistency

^b^Test re-test reliability

^c^Inter-rater reliability; Known groups–KG; Discriminant–Discrim.; Convergent–Converg.; Concurrent–Concur; predictive–Predict.

**Table 5:** Approaches to determining reliability and validity and the associated findings for newly developed tools for each study included in phase 2 analysis.

First author [citation] (n)	Reliability			Validity									
	IC^a^	TR	IR	Construct				Criterion					
				Structural	KG	Discrim.	Converg.	Concurrent	Predictive				
									Sens	Spec	PPV	NVP	ROC
**Brown** [45]^b^ (n = 186)	0.78–0.87			4 factors									
**Getting it Right Collaborative** [46] (n = 500)	0.88							MINI—22%	84% (74–91)	77% (71–83)	51%	95%	0.88 (85–92%)
**Esler** [54] (n = 35)	0.80							Semi-structured diagnostic interview—74%	70% (55–86)	78% (64–92)	58% (42–75)	86% (74–98)	Not reported
**Janca** [20] (n = 30)			0.56–1.0					Medical record—93%					
**Marley** [60] (n = 97)	0.89							GP Assessment based on DSM-IV and Australian GP Mental State Examination– 86%	83% (61–94)	87% (76–93)	68%	94%	Not reported
**Snodgrass** [62] (n = 219)	0.87			6 positive and 4 negative factors	Different scores across villages with different contexts		HSCL-10: -0.49 BSI: -0.33						
**Almeida** [56] (n = 250)	0.88							(n = 144) Psychiatric interview using ICD-10 and DSM-IV-TR criteria	78%	82%	39%	96%	0.88
**Brinckley** [50]^c^ (n = 6988)	0.89			1 factor		Self-report of lifetime doctor diagnosis of heart disease (12%)	Self-report of lifetime doctor diagnosis of depression (65%) and/or anxiety (57%) Self-report happiness in preceding 4 weeks (35%)	Self-report of lifetime doctor diagnosis of depression and/or anxiety	71% dep. 71% anx.^d^	68% dep. 65% anx.	22% dep. 20% anx.	4% dep. 5% anx.	Not reported
**Haswell** [51] (n = 184)	0.85^e^ ^–^ 0.89			K6+2 Single factor EES 2 factors S12 2 factors			K6 + EES: -0.48 K6 + S12: -0.45						
**Campbell** [53] (n = 210)	0.84–0.92f						EPDS and TAIHS = 0.47 antenatal (n = 24) 0.23 postnatal (n = 9)						
**Schlesinger** [57]^g^ (n = 175)	0.81	0.81 (n = 95)		1 factor			DASS anx: 0.62 DASS dep: 0.71 SRQ: 0.74	SRQ	83%	84%			

Abbreviations used in table: Internal consistency—IC; Test re-test–TR; Inter-rater—IR; Known groups–KG; Discriminant–Discrim.; Convergent–Converg.; Sensitivity–Sens: Specificity–Spec; Positive Predictive Value–PPV; Negative Predictive Value NPV; Receiver Operating Characteristics—ROC; Hopkins Symptom Checklist– 10 -HSCL-10; Bradford Somatic Index—BSI; Depression–Dep; Anxiety–Anx; Kessler Psychological Distress Scale– 6—K6; Emotional Empowerment Scales–EES; Empowerment scenarios–S12; Edinburgh Postnatal Depression Scale–EPDS; Townsville Aboriginal and Islander Health Service–TAIHS; Depression, Anxiety and Stress Scale–DASS; Self -report questionnaire -SRQ; Mini International Neuropsychiatric Interview–MINI.

^a^Where the internal consistency of more than one tool is reported a range is given.

^b^The tool used the aPHQ-9 and K6 but did not report the validity and reliability with the other scales.

^c^Convergent and divergent validity associated with very high levels of psychological distress according to MK-K5.

^d^Cut-off of 11 denotes categories used in population-level research not clinical indicator.

^e^Higher internal consistency was achieved with K6 + 2 (0.87) but with lower response rate (n = 141).

^f^This is the only tool that meets Nunnally’s [65] ≥ 0.90 threshold for internal consistency

^g^Only mental health screener reported here

**Table 6:** Quality of measurement properties for the determination of reliability and validity for each screening tool.

First author [citation]	Reliability		Validity					Total positive ratings
	Int. consis.	Inter-rater or test re-test	Content	Construct		Criterion		
				Struct.	Discrim. and/or converg.	Concur.	Predict^e^	
**aPHQ-9**								
Brown [44, 45]	+	0	+	?	0	0		2
Esler [54, 55]	+	0	+	0	0	+^a^	weak	3
Getting it Right Collaborative [46]	+	0	+	0	0	+	fair	3
**MK-K5**								
Brinckley [50]	+	0	+	+	+	?	weak	4
**EPDS**								
Campbell [53]	+	?	+	0^b^	0	0		2
**KMMS**								
Kotz [59] Marley [60] Carlin [58]	+	0	+	0	0	+	strong	3
**KICA-Dep**								
Almeida [56]	+	0	?	0	0	?	fair	1
**IRIS**								
Schlesinger [57]	+	+	0	-	+	?	strong	3
**GEMS**								
Haswell [51]	+	0	+	-	0	0		2
**HANAA**								
Janca [20]	0	-^c^	+	0	0	?		1
**PANAS**								
Snodgrass [62]	+	0	+	-	-^d^	0		2

Abbreviations used in table: Discriminant–Internal consistency–Int. consis.; Structural–Struct; Discrim.; Convergent–Converg; Concurrent–Concur; Predictive–Predict.

^a^Correlation between gold standard and modified tool not reported.

^b^Factor analysis is mentioned but a lack of *n* meant that it was not calculated.

^c^ Functioning scored 1.0 and substance use 0.70. Rated ‘–‘ because these were only two scores out of 10 that met the weighted Kappa ≥ 0.70 criterion.

^d^ Five different tools were used only two of them were correlated with the PANAS >0.50

^e^ DOR not included in the total positive ratings calculation as it was determined using another method.

Of the methods for determining tool reliability, internal consistency using Cronbach’s Alpha was the most reported (n = 10). All articles reported the findings of at least one type of validity. Content validity was the most reported (n = 9) and criterion validity was the second most reported (n = 7). Seven articles determined whether the newly developed tool demonstrated concurrent validity with an established tool or other relevant indicator [20, 46, 50, 54, 56, 57, 60]. Subsequently, six articles reported predictive validity [46, 50, 54, 56, 57, 60]. Three articles [46, 54, 56] predicted depression and the other three articles predicted both depression and anxiety [50, 57, 60]. The Getting it Right Collaborative [46] and Almeida and colleagues [56] predicted a current diagnosis of depression. The other articles [50, 54, 57, 60] did not report a timeframe for diagnosis. In addition, only three used a reference (gold) standard [54, 56, 60] which is the preferred method [36]. The MINI, which is a structured clinical interview, has not been included as a reference standard because it does not meet the definition for a reference/gold standard adopted by this study. Five articles reported construct validity with convergent validity being the preferred type [50, 51, 53, 57, 62]. Content validity determined using EFA [45, 51, 57, 62] or principal component analysis, [50] was also reported in five articles (see Table 5 below).

Further analysis determined whether the findings of reliability and validity for each study met the quality criterion for measurement properties (S2 Table). Table 6 below presents the findings of this analysis. Data has been organised according to the tool name as some development processes resulted in several related publications that reported different aspects of reliability and/or validity. In addition, the ratings and quality criteria identified in S2 Table have been applied to data in Table 6.

Internal consistency determined using Cronbach’s Alpha consistently achieved values that met the quality criterion of > 0.70 for a positive rating for all but one of the screening tools. The internal consistency of the HANAA [20] was not reported, which is not surprising given the qualitative nature of its approach. In contrast, inter-rater and test re-test reliability only met the quality criteria once [62] and did not meet the quality criteria in another [20]. However, there was indeterminable/no information available about this type of reliability reported across seven screening tools [44–46, 50, 51, 53–56, 58–60, 62].

Content validity achieved a positive rating for the development of all but two of the screening tools. Target population involvement in tool development was unable to be determined for the IRIS [57] or KICA-Dep [56]. In contrast, construct validity received a negative rating more often than all the other categories (n = 4) or was given a ‘no information available’ rating (?) (n = 14) for either or both sub-categories of structural and hypothesis testing.

The most indeterminable ratings (?) were allocated for criterion (concurrent) validity. This was because authors did not use a reference standard, or they used a less well recognised design or method. Three tools were rated ‘?’ because they did not use a reference standard [20, 50, 57]. For example, Brinckley et al., [50] used participants’ self-reported diagnosis of depression and/or anxiety by a GP to determine concurrent validity. The authors reported that this approach was a limitation. However, they also did not find a correlation ≥ 0.70 with participants’ self-reports, which also meant that their findings did not meet the ‘+’ rating on this criterion.

In contrast, Almeida et al., [56] did not conduct a psychiatric interview to determine concurrent validity with all participants, only with those that scored above 9 on the KICA-Dep. This approach meant that they were unable to ‘rule-out’ false negatives from participants who scored below their cut-off. This is a design issue that is pertinent to the determination of the predictive validity for the KICA-Dep.

When considering the quality of the measurement properties for each of the screening tools, Brinckley and colleagues [50] received the most positive ratings with four out of a possible six for the adapted K5 –MK-K5. Conversely, Snodgrass and colleagues [62] received the most negative ratings (n = 2) and only 2 positive ratings out of six for their PANAS. Furthermore, seven author groups [20, 44–46, 53–56, 58–60] were rated ‘no information available’ (0) on at least three of the measurement categories. These ratings were clustered around reliability (inter-rater and test re-test) and construct validity. Of the three types of validity, construct validity is the least investigated in this data set and when it was, poor quality outcomes were more likely.

Using the classification levels for predictive validity [37], Esler and colleagues [54] adaption of the PHQ-9 and Brinckley et al., of the K5 [50] were weak, adaptation of the PHQ-9 [46] and the KICA-Dep [56] fair, and the adaptation of the EPDS to the KMMS [60] and development of the IRIS [57] were strong. Data is available in S3 Table.

Tool acceptability (face validity) is an important component of determining its validity. Acceptability was only reported in four articles [46, 50, 56, 60] and this was done to varying degrees. For example, the Getting it Right Collaborative [46] specifically asked about acceptability. Participants identified that overall, they found the aPHQ-9 acceptable, although 8% of participants identified that some questions were a bit too personal. Likewise, in Marley et al., [60] participants also completed qualitative questionnaire about the acceptability of the KMMS, with 44%. reporting that completing the KMMS was a positive experience. In contrast, Brinckley et al., [50] assumed acceptability based on response rates and missing values. Similarly, Almeida et al., [56] indicated that the KICA-Dep was well accepted by their participants but did not provide any detail about how this was determined.

### Is there an overarching development approach? Synthesis of qualitative and quantitative findings

The answer to this question is two-fold. First, yes, conceptually there is an overarching development approach that was advocated by many authors [35, 41, 39–42] and encapsulated in the conceptual model used in this review.

Second, this review found that relatively few researchers followed the whole developmental approach suggested in the literature. The findings of phase one (qualitative) indicated that steps involved in developing the scale (pre-test questions; pilot; reduce number of questions and determine domains) were reported the least. In contrast, steps required to assess the scale (examine domains, assess reliability, validity and determine clinical utility) were completed by most developers, with reliability and validity routinely assessed.

The results of phase two (quantitative) analysis found that internal consistency was most often reported and achieved the quality criterion. Conversely, validity was less rigorous. Of the three different types of validity, content validity was most often reported, which was reassuring given the importance of involving the target population in both development and in determining acceptability [66]. Criterion validity was the second most reported type of validity, with concurrent validity being determined most often. Most authors subsequently determined predictive validity so that the tool could be used in a clinical setting to support referral. Construct validity was reported least often, which was of concern, given this has been described as one of the most important types of validity [35]. In the case of cross-cultural adaptation of standard tools, it is important that it be determined especially if questions have been changed which may alter the construct(s) for which the original tool was developed.

## Discussion

This review was focussed on answering the research question: What qualitative and quantitative approaches are used to develop new tools to screen for distress in Indigenous adults globally?

The conceptual framework identified that the first two stages of tool development: 1) develop questions; and 2) develop scale, were less frequently reported in this data set. These two stages use both qualitative and quantitative methods with mainly qualitative methods used for the first stage and mainly quantitative methods in stage two. The third stage of tool development, assessing validity and reliability, were routinely reported, and typically used quantitative methods. When assessing tool reliability and validity, developers routinely presented findings related to internal consistency and content validity. Conversely, repeatability (inter-rater and/or test re-test), acceptability (face validity), criterion and construct validity were reported less often.

Findings of note are related to construct and criterion validity. Construct validity was only determined in 5% of studies included in this review. This is an issue given the importance [35, 66–68] of determining construct validity when cross-culturally adapting tools where semantic, idiomatic, experiential, and conceptual equivalence between the original and adapted tool need to be maintained [41, 42]. To support decolonising psychology, perhaps tool developers should follow the example of authors of tools in category four [20, 62] by grounding their constructs in Indigenous peoples’ holistic conceptualisation of wellbeing.

In relation to criterion validity, 82% of tools were developed with the intent of being used in a clinical setting. Consequently, criterion validity (both concurrent and predictive) should be determined. Unfortunately, determining concurrent validity with a valid reference standard is not currently possible for Indigenous peoples because there are no appropriate diagnostic measures. According to Kisley et al., [69] the applicability of diagnostic measures that are derived from the ICD or DSM criteria for Indigenous populations is unclear. Black et al., [70] also identified that the CIDI was not valid for Australian Indigenous peoples. A point that Basit et al., [71] agreed with. Additionally, Black and colleagues [70] suggested that the validity of clinical interviews relied on the cultural competence of the practitioner. Validating an appropriate diagnostic approach for Indigenous peoples is a significant gap in the literature. Implications for tool developers are that they should carefully consider the screening tool’s intended use so that they can plan (time and cost) for the number of steps needed for validation.

### Number of steps necessary to validate a new screening tool

The number of steps needed to validate a new screening tool is dependent on the type of tool being developed. Following the recommendations of Gone [17], Dudgeon et al., [4, 13, 14] new tools should adopt the approach of decolonising psychology and be developed with Indigenous peoples using their holistic conceptualisation of wellbeing. Utilising the conceptual model (Fig 1) we have identified that a total of nine steps should be completed. Two steps to develop questions (identify domain and associated questions and establish content validity), all steps to develop (4) and assess (3) the scale. However, if a tool is being adapted, which is not recommended due to issues arising with construct validity, an additional step is required to translate and cross-culturally adapt the original tool. Consequently, ten steps should be completed.

If a tool is a questionnaire and uses a Likert type scale, provides a score, cut-off points and needs to be predictive it will need different tests of reliability and validity than one that uses a conversational approach. For example, contrast the different reliability and reliability tests for the adapted PHQ-9 [54] and the HANNA [20]. The adapted PHQ-9 was designed to be used in a clinical setting, uses a Likert scale, has cut-off points, and needs to be able to predict subsequent diagnosis. A test of internal consistency is necessary as well as determinations of content, construct, and criterion validity. In contrast, the HANNA, which is also designed to be used in a clinical setting, adopts a conversational approach using a dichotomous key indicating whether there is a problem or no problem. This approach benefits from a determination of inter-rater reliability. The absence of scoring means that there is no cut off and predictive validity cannot be determined. However, concurrent validity should by determined by using an appropriate diagnostic approach. Although it has not been reported to date HANAA would benefit from determinations of construct validity.

### Limitations

There a several limitations of this review. First, only English language publications were reviewed. This was because the authors are only fluent in the English language and did not have access to funds to pay for translation services.

Second, the data set was limited to publications since 2000. However, on reviewing the publication dates of this data set, publications began appearing in 2007, with a consistent rise since then which suggests relevant papers have been included. In support of this, a related scoping review [12] examining the use of depression and anxiety screening tools with Indigenous peoples globally, searched databases from inception and did not locate any further publications relevant to this review.

Third, sixty percent of the articles were located using citation searches. This indicates that, despite the assistance of a relevant librarian, the database searches were ineffective, possibly due to the keywords selected by the authors. Ali and colleagues [37] also identified this limitation in their systematic review of the validation of screening tools used in low- and middle-income countries. For this review, it may also be indicative of the shift in terminology towards wellbeing and away from mental health for Indigenous peoples.

Finally, the absence of known groups and predictive validity quality criteria for measurement properties (S1 Table) meant that we were unable to determine the quality of these outcomes for this SMSR.

### Implications

The implications of this review for the development of new tools to screen for distress in Indigenous peoples globally is particularly salient for practice and provides several avenues for future research. In practice, the need for appropriate, valid, and reliable tools that screen distress in Indigenous peoples has not diminished and aligns with the need to decolonise psychology. Conversely, demand may well have increased over the past few years, given that the World Health Organisation reported rates of common mental health conditions such as depression have increased by more than 25% in the first year of the COVID-19 pandemic [72]. Equitable access to support for people in distress relies firstly on screening using a valid and reliable tool. However, distress is usually screened using tools developed using the Western biomedical paradigm which may be inappropriate for Indigenous peoples [12]. The impact of the use of non-validated screening tools with Indigenous peoples may result in inequitable access to mental health care. It is also important that health services endorse the use of screening tools that have undergone adequate reliability and validity testing, so clinicians have confidence in the accuracy of their assessments.

Whilst a substantial body of work has already been completed in Australia in particular, the construct validity of a number of these tools is yet to be determined. Future research could focus on determining the construct validity for the populations that they are routinely being used with. This would enhance the body of evidence which could in turn support the confidence of practitioners to use them.

The quality of the studies included in this review was varied, with MMAT score rating of most studies being between 0–3 out of 5. In most studies, this was due to a lack of a research question, which in the MMAT, has implications for the subsequent evaluation of the study design. In addition, in studies that reported validation of their tools, most had issues with determining construct and criterion validity. Given the importance of construct validity this is an issue. In future researchers could consider the use of a study design checklist such as one provided by COSMIN [73] to reduce the risk of bias [34]. Although the COSMIN checklist is designed for patient reported outcomes, it could be adapted for study designs focussed on developing new screening tools. This type of approach to study design has been advocated for in the past [34].

Given that most studies in this review were conducted in Australia, the lack of an appropriate diagnosis for First Nations peoples [70] has been raised as an issue that impacts establishing criterion (concurrent) validity of screening tools. Future research focusing on establishing the validity of a diagnostic tool and/or approach for Australian First Nations peoples may address this issue. In the interim, tool developers should follow the suggestion of Black et al., [70] in relation to the cultural competence of practitioners and use of a reporting framework is recommended. In addition, researchers from other countries may consider whether an appropriate diagnostic approach exist in their context. If an appropriate valid diagnostic approach does not exist, then they need to consider the impact of its absence on their attempt to validate their screening tool.

## Conclusion

This SMSR examined the extent of the literature related to approaches for developing new tools to screen for distress in Indigenous peoples globally. Overall, many studies did not take all the steps on the theoretical path, demonstrated in the conceptual model (Fig 1), to develop new tools. Studies conform to the methods that support each stage: qualitative, mixed, or quantitative and quantitative to 1) develop questions, 2) develop scale and 3) assess scale, respectively. However, they missed steps in the develop questions and develop scale stages. Furthermore, they collapsed steps needed to 2) develop scale and 3) assess scale. This may be due to short time frames and the associated resourcing associated with developing new tools.

Most studies completed steps associated with 3) assess scale, which included validation, but many tools did not exhibit construct validity and/or criterion validity. Failing to determine construct validity presents an issue because most of the tools were cross-culturally adapted from pre-existing standard tools. As construct validity demonstrates the theoretical relationship between questions in the tool and the underlying construct that they intend to measure, changing and adding questions may change this relationship. Over half of the studies provided findings of criterion validity by assessing concurrent validity with a reference standard. However, most did not use the reference standard for diagnosis.

### Summary of recommendations

Implications of the findings suggest that clinicians and researchers should consider whether the tools that they are using to screen for distress in Indigenous peoples are valid for the population that they are being used with. This includes investigating whether tools have been subject to rigorous validation and being aware where more work is required. Researchers and clinicians also need to be cognisant that previously validated tool(s) may not be valid in their context.

Future research in this area focusing on continuing to strengthen validity evidence of tools that have already been cross-culturally adapted, such as the aPHQ-9, KMMS, MK-K5 and KICA-Dep is warranted. Tools such as the HANAA, which originated through work conducted with Australian Aboriginal people appears promising and further validation would be worthwhile. To support the criterion validity of these screening tools, a culturally valid reference standard for diagnosis of Aboriginal and Torres Strait Islander peoples would be ideal but is currently lacking. Until developed, researchers and clinicians working in this area need to be cognisant of the need for culturally competent practitioners to make diagnoses and following the guidance of Black et al., [70] their level of competence should be reported in publications.

## Supporting information

S1 Checklist (DOCX)

S1 File Full search strategies for each database. (PDF)

S1 Table Quality criteria for measurement properties. (PDF)

S2 Table Correlation between MMAT and number of steps taken to develop the screening tool. (PDF)

S3 Table Values for weighted diagnostic odds ratio. (PDF)
