# Supplementary material for: Approaches to the development of new screening tools that assess distress in Indigenous peoples: A systematic mixed studies review
Source: PLoS One. 2023 Sep 8;18(9):e0291141. doi: 10.1371/journal.pone.0291141 (PMC10490875; doi:10.1371/journal.pone.0291141)
Supplement: S1 File — (PDF) [file pone.0291141.s002.pdf]

Supplementary File 1

Systematic review searches
(Note: search strings vary according to different databases)

All databases searched between 22 sept – 5 Oct 2021 and again on 6 March 2023

Medline

*Indigenous Peoples/

("first nations" or "first peoples" or aboriginal or indigenous).mp. [mp=title, abstract, original title, name of substance word, subject heading word, floating sub-heading word, keyword heading word, organism supplementary concept word, protocol supplementary concept word, rare disease supplementary concept word, unique identifier, synonyms]

*Mental Health/

("social and emotional wellbeing" or SEW or SEWB).mp. [mp=title, abstract, original title, name of substance word, subject heading word, floating sub-heading word, keyword heading word, organism supplementary concept word, protocol supplementary concept word, rare disease supplementary concept word, unique identifier, synonyms]

limit to (english language and yr="2000 -Current" and "all adult (19 plus years")

(screen* or tool* or measure* or evaluat*).mp. [mp=title, abstract, original title, name of substance word, subject heading word, floating sub-heading word, keyword heading word, organism supplementary concept word, protocol supplementary concept word, rare disease supplementary concept word, unique identifier, synonyms]

PsychINFO

su("indigenous people*" OR "first nations" OR "first peoples" OR aboriginal OR indigenous) AND su("mental health" OR "social and emotional wellbeing" OR SEW*) AND su(measure* OR tool* OR evaluat*) NOT ab(child* OR youth OR "young person" OR adolesc* OR student OR infant* OR school)

Date: From January 2000 to March 2023

Language
English
Age group
Adulthood (18 Yrs & Older)

CINAHL

S1 MW "Indigenous peoples"

S2 MW "Indigenous peoples" OR "first nations" OR Aboriginal OR "first peoples" OR Maori

S3 (MW “Indigenous peoples” OR “first nations” OR Aboriginal OR “first peoples” OR Maori AND (S1 AND S2)

S4 (MH “Indigenous peoples+”)

S5 (MH “First Nations of Australia+”) OR (MH “Aboriginal Canadians+”) OR (MH “Indigenous peoples +”) OR (MD Native Americans+”)

S6 S4 AND S5

S7 (MH “psychological tests+”)

S8 (assessment OR evaluation OR tool OR measure)

S9 (assessment OR evaluation OR tool OR measure) AND (S7)

S10 (assessment OR evaluation OR tool OR measure) AND (S7 OR S8) AND (S6)

S11 (MM “Mental Health”)

S12 “mental health” OR social and emotional wellbeing” OR SEW*

S13 (“mental health” OR social and emotional wellbeing” OR SEW*) AND (S11 OR S12)

S14 ((“mental health” OR social and emotional wellbeing” OR SEW*)) AND (S10 AND S13)

Limiters – Published date 20000202 – 20230306

Expanders – apply equivalent subjects

Narrow by language – English

Narrow by subject age – all adult

EMBASE

*indigenous people/

("first nations" or "first people" or aboriginal or indigenous).mp. [mp=title, abstract, heading word, drug trade name, original title, device manufacturer, drug manufacturer, device trade name, keyword heading word, floating subheading word, candidate term word]

("social and emotional wellbeing" or SEW or SEWB).mp. [mp=title, abstract, heading word, drug trade name, original title, device manufacturer, drug manufacturer, device trade name, keyword heading word, floating subheading word, candidate term word]

*mental health/

(screen* or tool* or measure* or evaluat*).mp. [mp=title, abstract, heading word, drug trade name, original title, device manufacturer, drug manufacturer, device trade name, keyword heading word, floating subheading word, candidate term word]

limit to (english language and exclude medline journals and yr="2000 -Current" and adult <18 to 64 years>

Emcare

Ovid Emcare

*indigenous people/

("first nations" or "first peoples" or aboriginal or indigenous).mp. [mp=title, abstract, heading word, drug trade name, original title, device manufacturer, drug manufacturer, device trade name, keyword]

*mental health/

("social and emotional wellbeing" or SEW or SEWB).mp. [mp=title, abstract, heading word, drug trade name, original title, device manufacturer, drug manufacturer, device trade name, keyword]

(screen* or tool* or measure* or evaluate*).mp. [mp=title, abstract, heading word, drug trade name, original title, device manufacturer, drug manufacturer, device trade name, keyword]

limit to (english language and yr="2000 - 2023" and adult <18 to 64 years>)

SCOPUS

((((TITLE-ABS-KEY (_"indigenous peoples"_ OR _"first nations"_ OR _"first peoples"_ OR _aboriginal_ OR _indigenous_)) AND (TITLE-ABS-KEY (_"social and emotional wellbeing"_ OR _sew*_ OR _"mental health"_)) AND (TITLE-ABS-KEY (_screen*_ OR _tool*_ OR _measure*_ OR _evaluat*_))) AND NOT (_pharma*_ OR _biol*_ OR _environ*_ OR _covid19_)) AND NOT (_child*_ OR _'young_ AND _person'_ OR _infant*_ OR _adolesc*_ OR _school*_ OR _youth_)) AND (LANGUAGE (_english_) AND PUBYEAR > _1999_)
