# Supplementary material for: Approaches to the development of new screening tools that assess distress in Indigenous peoples: A systematic mixed studies review
Source: PLoS One. 2023 Sep 8;18(9):e0291141. doi: 10.1371/journal.pone.0291141 (PMC10490875; doi:10.1371/journal.pone.0291141)
Supplement: S1 Table — (PDF) [file pone.0291141.s003.pdf]

S1 Table. Quality criteria for measurement properties (adapted from Terwee et al., [30] and Schellingerhout et al., [31])

Property	Rating	Quality criteria
Reliability		
Internal consistency[a]	+	(Sub)scale unidimensional AND Cronbach's alpha(s) ≥ 0.70
	?	Dimensionality not known OR Cronbach's alpha not determined
	-	(Sub)scale not unidimensional AND Cronbach's alpha(s) < 0.70
	0	No information found on internal consistency
Interrater or test-retest	+	ICC/weighted Kappa ≥ 0.70<br>OR Pearson's *r* ≥ 0.80
	?	Neither ICC/weighted Kappa nor Pearson's *r* determined
	-	ICC/weighted Kappa < 0.70 OR Pearson's *r* < 0.80
	0	No information found on reliability
Validity		
Content	+	The target population considers all items in the tool to be relevant AND considers the tool to be complete
	?	No target population involvement
	-	The target population considers all items in the tool to be irrelevant OR considers the tool to be incomplete
	0	No information found on content validity
Construct		
Structural	+	Factors should explain at least 50% of the variance
	?	Explained variance not mentioned
	-	Factors explain < 50% of the variance
	0	No information found on structural validity
Convergent and discriminant	+	(Correlation with an instrument measuring the same construct ≥ 0.50 OR at least 75% of the results are in accordance with the hypotheses) AND correlation with related constructs is higher than with unrelated constructs
	?	Solely correlations determined with unrelated constructs
	-	Correlation with an instrument measuring the same construct < 0.50 OR < 75% of the results are in accordance with the hypotheses OR correlation with related constructs is lower that with unrelated constructs
	0	No information found on hypothesis testing
Criterion		
Concurrent	+	Convincing arguments that gold standard is "gold" AND correlation with gold standard ≥ 0.70
	?	No convincing arguments that gold standard is 'gold' OR doubtful design or method[b]
	-	Correlation with gold standard <0.70, despite adequate design and method
	0	No information found on concurrent validity

Table legend: Rating + = positive rating; ? = indeterminate rating; - negative rating; 0 = no information available.

[a]It is noted that Cronbach's alpha(s) ≥ 0.70 is low for tools designed for clinical use. Alpha levels for clinical tool should be ≥ 0.90 [66]

[b]Doubtful design or method = lacking a clear description of design or methods of the study, sample size smaller than 50 participants (should be at least 50 in each subgroup), or any important methodological weakness in design or execution of study.
