# Supplementary material for: Approaches to the development of new screening tools that assess distress in Indigenous peoples: A systematic mixed studies review
Source: PLoS One. 2023 Sep 8;18(9):e0291141. doi: 10.1371/journal.pone.0291141 (PMC10490875; doi:10.1371/journal.pone.0291141)
Supplement: S2 Table — (PDF) [file pone.0291141.s004.pdf]

S2 Table. Correlation between the MMAT and number of steps taken to validate the screening tool.

First author [citation]	MMAT quality score	Mean quality where needed	Dichotomous MMAT score (0 – 2 = low; 3-5 = high)	Steps
Almeida [57]	1		1	4
Brinckley [5]	3		2	3
Brown [45]	3	2	2	11
Brown [46]	0			
Campbell [54]	2		1	3
Carlin [59]	3	4	2	8
Carlin [53]	3			
Esler [56]	3	2		4
Esler [55]	1			
Farnbach [48]	2			
Getting it Right Collaborative [47]	2			
Gomez Cardona [50]	0			1
Haswell [52]	2			4
Janca [21]	3	2		7
Janca [62]	1			
Kotz [60]	5			
Marley [61]	3			
Schlesinger [58]	0			5
Snodgrass [63]	2			8

Where tool development was reported over several publications a mean MMAT was calculated. Publications grouped together are highlighted in the same colour. Where the calculated sum included a fraction it was rounded up to the nearest whole number. To enable correlation to be calculated as MMAT scoring was ordinal it was converted to dichotomous. Therefore, MMAT scores 0 – 2 were coded 1 (low) and scores 3 – 5 were coded 2 (high)

Correlations

			Quality	Steps
Spearman's rho	Quality	Correlation Coefficient	1.000	.351
		Sig. (2-tailed)	.	.290
		N	11	11
	Steps	Correlation Coefficient	.351	1.000
		Sig. (2-tailed)	.290	.
		N	11	11

Very low sample size p =0.290

Spearman’s Rho =
