# Supplementary material for: Approaches to the development of new screening tools that assess distress in Indigenous peoples: A systematic mixed studies review
Source: PLoS One. 2023 Sep 8;18(9):e0291141. doi: 10.1371/journal.pone.0291141 (PMC10490875; doi:10.1371/journal.pone.0291141)
Supplement: S3 Table — (PDF) [file pone.0291141.s005.pdf]

S3 Table. Values for weighted diagnostic odds ratio

First author [citation] (n)	Predictive					
	Sens	Spec	DOR	PPV	NVP	ROC
**Brown** [46] (n = 186)						
**Getting it Right Collaborative** [47] (n = 500)	84% (74-91)	77% (71-83)	18.10	51%	95%	0.88 (85-92%)
**Esler** [55] (n = 35)	70% (55-86)	78% (64-92)	8.32	58% (42-75)	86% (74-98)	Not reported
**Janca** [21] (n = 30)						
**Marley** [61] (n = 97)	83% (61-94)	87% (76-93)	34.85	68%	94%	Not reported
**Snodgrass** [63] (n = 219)						
**Almeida** [57] (n = 250)	78%	82%	16.85	39%	96%	0.88
**Brinckley** [51] (n = 6988)	71% dep. 71% anx.	68% dep. 65% anx.	5.19 dep. 4.60 anx.	22% dep. 20% anx.	4% dep. 5% anx.	Not reported
**Haswell** [52] (n = 184)						
**Campbell** [54] (n = 210)						
**Schlesinger** [58] (n = 175)	83%	84%	25.68			

Abbreviations used in table: Sensitivity – Sens: Specificity – Spec; Positive Predictive Value – PPV; Negative Predictive Value NPV; Receiver Operating Characteristics - ROC
